# Antinociceptive effects of green synthesized copper nanoparticles alone or in combination with morphine

**DOI:** 10.1016/j.amsu.2019.12.006

**Published:** 2020-01-11

**Authors:** Hossein Mahmoudvand, Mojtaba Khaksarian, Katrin Ebrahimi, Sima Shiravand, Sareh Jahanbakhsh, Massumeh Niazi, Sedigheh Nadri

**Affiliations:** a Razi Herbal Medicines Research Center, Lorestan University of Medical Sciences, Khorramabad, Iran; b Department of Biology, Payame Noor University, Tehran, Iran; c Student Research Committee, Lorestan University of Medical Sciences, Khorramabad, Iran; d Department of Anesthesiology, Lorestan University of Medical Sciences, Khorramabad, Iran; e Department of Biochemistry, Lorestan Uniersity, Khorramabad, Iran

**Keywords:** Nanoparticles, Copper, Tail flick test, *Capparis spinosa*, BALB/c mice

## Abstract

**Objective:**

The aim of this study was to evaluate the antinociceptive effect of biosynthetic copper nanoparticles from aqueous extract of *Capparis spinosa* fruit.

**Methods:**

In this study, green synthesis of copper nanoparticles (CuNPs) was performed using *C. spinosa* extract according to the method described previously. The synthesized CuNPs were characterized using the UV–vis spectroscopy, Fourier transforms of infrared (FTIR), scanning electron microscopy (SEM), and energy-dispersive X-ray (EDX). The antinociceptive effect of CuNPs was evaluated by tail-flick, hot-plate, and rotarod tests following the oral administration of mice with CuNPs at the concentrations of 25, 50, and 75 mg/kg for two weeks.

**Results:**

The obtained maximum peak at the wavelength of 414 nm demonstrated the biosynthesis of the copper nanoparticles. SEM approved the particle size of CuNPs between 17 and 41 nm. The statistical analyses of the data of hot plate and tail-flick tests showed the potent analgesic effect of biosynthetic CuNPs. In this regard, the antinociceptive effect of at the doses of 75 mg/kg and 25 mg/kg plus morphine was significantly higher in comparison with the control group receiving morphine alone (P < 0.05). No significant (p > 0.05) difference was observed after the administration of CuNPs at the doses of 25, 50, and 75 mg/kg in the sensory-motor test.

**Conclusion:**

The present investigation demonstrated the analgesic effects of CuNPs especially in combination with morphine. These findings can provide a new strategy for producing new antinociceptive medications in the future.

## Highlights

• We evaluated the antinociceptive effects of green synthesized copper nanoparticles.
• The antinociceptive effect of CuNPs was evaluated by tail-flick, hot-plate and rotarod test.
• Hot plate and tail flick tests showed the potent analgesic effect of biosynthetic CuNPs.
• No significant difference was observed after administration of CuNPs in sensory motor test.
• The results showed the analgesic effects of CuNPs especially in combination with morphine.

## 1 Introduction

Pain is a complex mechanism that is caused by harmful stimuli in nature and is one of the most common symptoms of discomfort in individuals [1]. This phenomenon leads to changes in both the peripheral and central nervous systems so that sensory information is first transmitted to the spinal cord through the peripheral nervous system and then sent to higher centers in the central nervous system for perception and final interpretation [2,3]. Although opioid drugs today are widely used to relieve pain, their use can lead to major side effects such as a reduced threshold of pain tolerance, i.e., hyperalgesia, and physical and psychological dependence [4].

Nanotechnology, which plays a very important role in modern researches, is the application of science to control material at the molecular level [5]. Nowadays, due to the costly and dangerous physical and chemical techniques, to produce metal nanoparticles (NPs), biosynthetic methods are used because of producing no toxic substances in the environment (green synthesis) [5]. Copper (Cu) is one of the most useful elements in medical sciences regarding numerous therapeutic effects such as anti-inflammatory, anticancer, analgesic, and antimicrobial effects [6].

Previous studies indicate the synthesis, characterization, and biological activities of copper nanoparticles and copper nanocrystals in different ways, however, the green synthesis of copper nanoparticles has been little attention in studies [[7], [8], [9], [10], [11], [12], [13], [14]]. The present investigation was aimed to synthesize the green synthesis of CuNPs using the aqueous extract of *C. spinosa* and evaluate the acute and chronic antinociceptive effects of these nanoparticles in BALB/c mice model.

## 2 Materials and methods

### 2.1 Collection of plant materials

The fruits of *C. spinosa* were collected from the rural areas of Kohdasht district, Lorestan Province located in western Iran in August 2017. Then, the plant materials were identified by a botanist in Herbarium of Agriculture and Natural Resource Research Center (ANRRC), Khorramabad, Iran. A voucher specimen was deposited at herbarium of ANRRC (N0. 22785).

### 2.2 Preparation of aqueous extract

Three hundred g of plant were extracted by percolation procedure by means of methanol (80%) for three days in room temperature. To remove the artifacts the extract was passed through filter paper (Whatman No.3, Sigma, Germany). Extract was concentrated in vacuum at 50 °C by means of a rotary evaporator and stored in the refrigerator [15].

### 2.3 Characterization of CuNPs

#### 2.3.1 Green synthesis of copper nanoparticles

In the present investigation, the green synthesis of CuNPs was performed according to the method described by the present authors. Briefly, 75 ml of the obtained extract was added to 100 ml 0.01 M copper sulfate solution; after stirred it was kept at 60 °C for one day. In the next step, to remove all impurities it was centrifuged twice at the 12,000 rpm for 20 min. The nanoparticles have been prepared when the color of the solution changed from green to amber yellow. The synthesized nanoparticles were dried in the oven at 60 °C for the more analyses [16,17].

#### 2.3.2 UV–Vis spectroscopy analysis

When the copper ions turned into the copper nanoparticles, the process was confirmed by the surface plasmon resonance (SPR) of the copper nanoparticles. So that, 0.3 ml of the NPs solution was diluted with 3 ml of normal saline, and were assessed by UV–Vis spectrum analysis by means of a spectrophotometer device (JENWAY 6405) in the range of 300–700 nm [18].

#### 2.3.3 Fourier transform infrared spectroscopy

FTIR was performed in the range of 400–4000 and with the resolution of 1–4 cm on the mixture of the obtained NPs along with the KBr granules with the ratio of 1–100 after becoming tablets [19].

#### 2.3.4 Scanning electron microscope (SEM)

The characteristics including size and morphology of the obtained CuNPs was studied by means of a scanning electron microscopy (Mira3, Made in Czech) with 15 kv, magnification of x10, and resolution of 1 nm.

### 2.4 Animals

From the Tehran Pasteur Institute were purchased 84 male BALB/c mice with a weight of 25–30 g and kept with same light-dark cycles (12:12-h), while the room temperature was (22 ± 2 °C) and enough food and water were provided for mice. They were placed in laboratory conditions 30 min before the start of the experiment.

This study was carried out in strict accordance with the recommendations in the Guide for the Care and Use of Laboratory Animals of the National Institutes of Health. Moreover, The protocol of the study was approved by the Ethics Committee of Lorestan University of Medical Sciences, Lorestan, Iran (No. 2018/A-10-1633-4).

### 2.5 Tail flick test

Tail flick test is focusing the light burning on the middle one-third of the animal's tail. In this experiment, 30 mice in five groups (6 mice in each group) were treated with different concentrations of CuNPs (25, 50, and 75 mg/kg), morphine (positive control) as well as morphine along with 25 mg/kg of CuNPs in tail flick test and their termal pain threshold was evaluated. Light intensity of the tail flick apparatus (Sparco, Iran) was adjusted to make a 2–4 s latency time in the intact animal. A cutoff time of 10 s was considered to prevent any possible tissue damage. Latency time was recorded thrice with 15-min interval for each set of the tail flick test; the mean was considered as a thermal pain threshold (tail flick latency) (20).

### 2.6 Hot plate test

The plate temperature of the device was set to 55 ± 0.2 °C for evaluation of pain sensitivity of treated mice with CuNPs (25, 50, and 75 mg/kg), morphine (positive control) as well as morphine + 25 mg/kg of CuNPs. The desired apparatus contained a plate with the diameter of 19 cm and a Plexiglas wall with height of 30 cm (LE710 model, Lsi LETICA, Spain). The interval between the start of the experiment and the licking front paw or jumping measured as reply time to thermal pain (maximum cutoff was considered 60 s) (20).

### 2.7 Rotarod test

To evaluate the motor coordination in mice we used the rotarod test. Briefly, mice were educated to keep for 3 min on a rolling rod (3 cm, diameter) rotating at 8 rpm. Then three groups (six mice in each) were administrated by the various doses of CuNPs (25, 50, and 75 mg/kg) and one group received normal saline as negative control. Finally after administration CuNPs and normal saline, animals were placed on the rolling rod and the number of falls experienced by the mice during the procedure (3 min) was registered [20].

### 2.8 Statistical analysis

SPSS software version 17.0 (SPSS Inc., Chicago, IL, USA) was used to analyze the obtained data. One-way ANOVA test as well as Tukey's post-hoc test was used to assess the difference between experimental groups. And finally P < 0.05 was considered statistically significant.

## 3 Results

### 3.1 Characteristics of CuNPs

#### 3.1.1 UV–Vis spectrum analysis

**Fig. 1:**
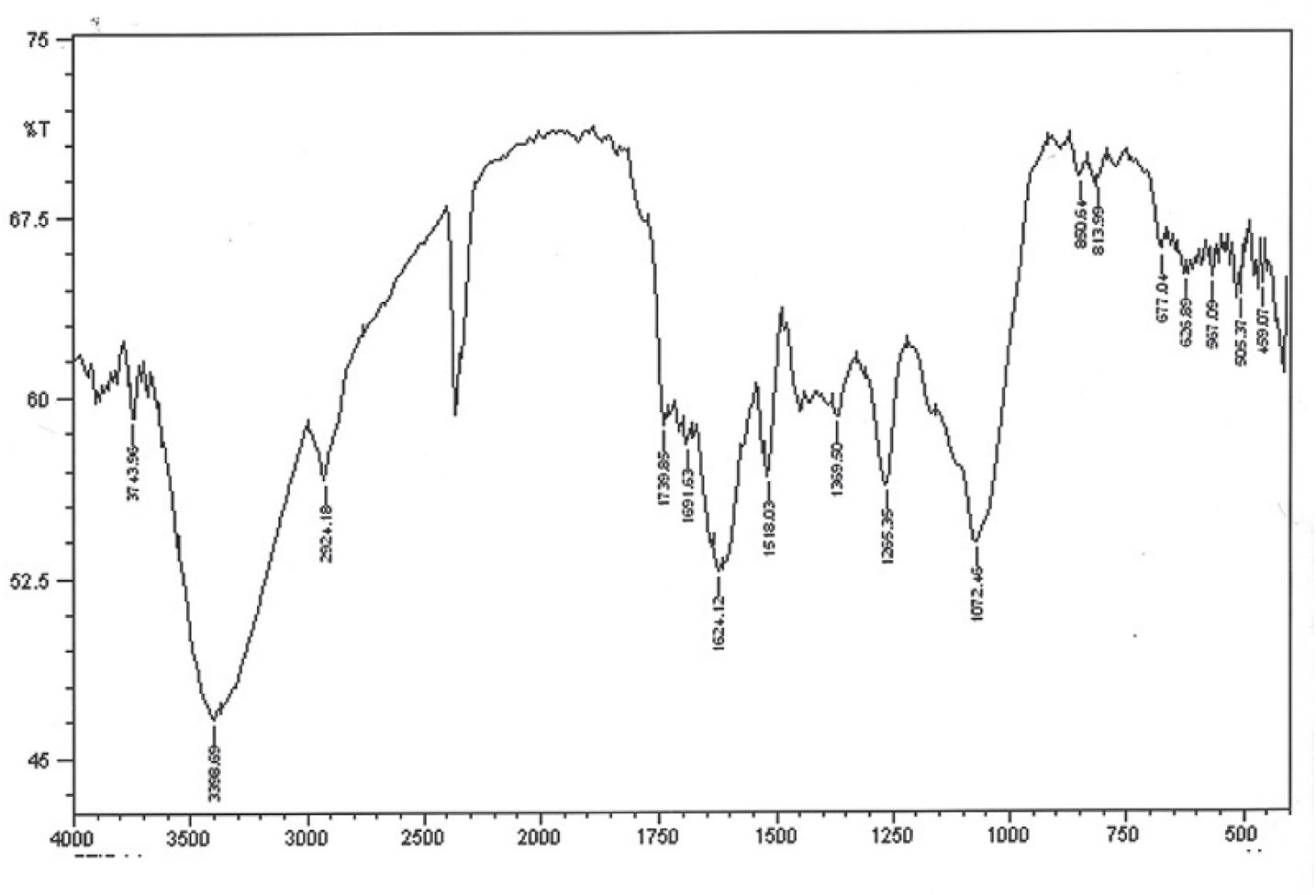
EDX to check the presence of copper element.

**Fig. 2:**
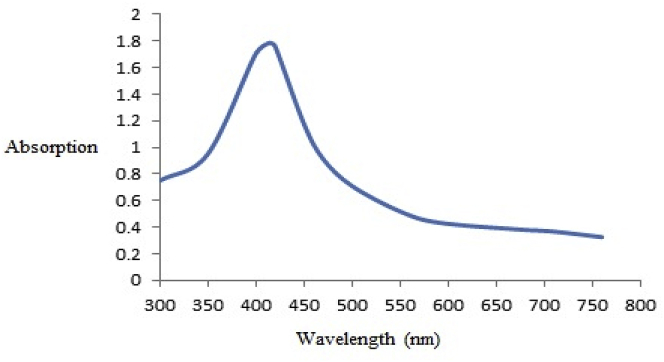
The absorption spectrum of synthesized copper nanoparticles.

The highest peak of the synthesized CuNPs was observed in the zone of 414 nm. The metallic copper existence was proved by EDX analysis (Fig. 1). The copper nanoparticles at 1 KeV revealed a sorptive peak, indexing for metallic nanoparticles of copper. The obtained findings demonstrate that at the wavelength of 414 nm, the characteristic of the resonance band of the surface plasmon happened for CuNPs (Fig. 2).

#### 3.1.2 FTIR analysis

**Fig. 3:**
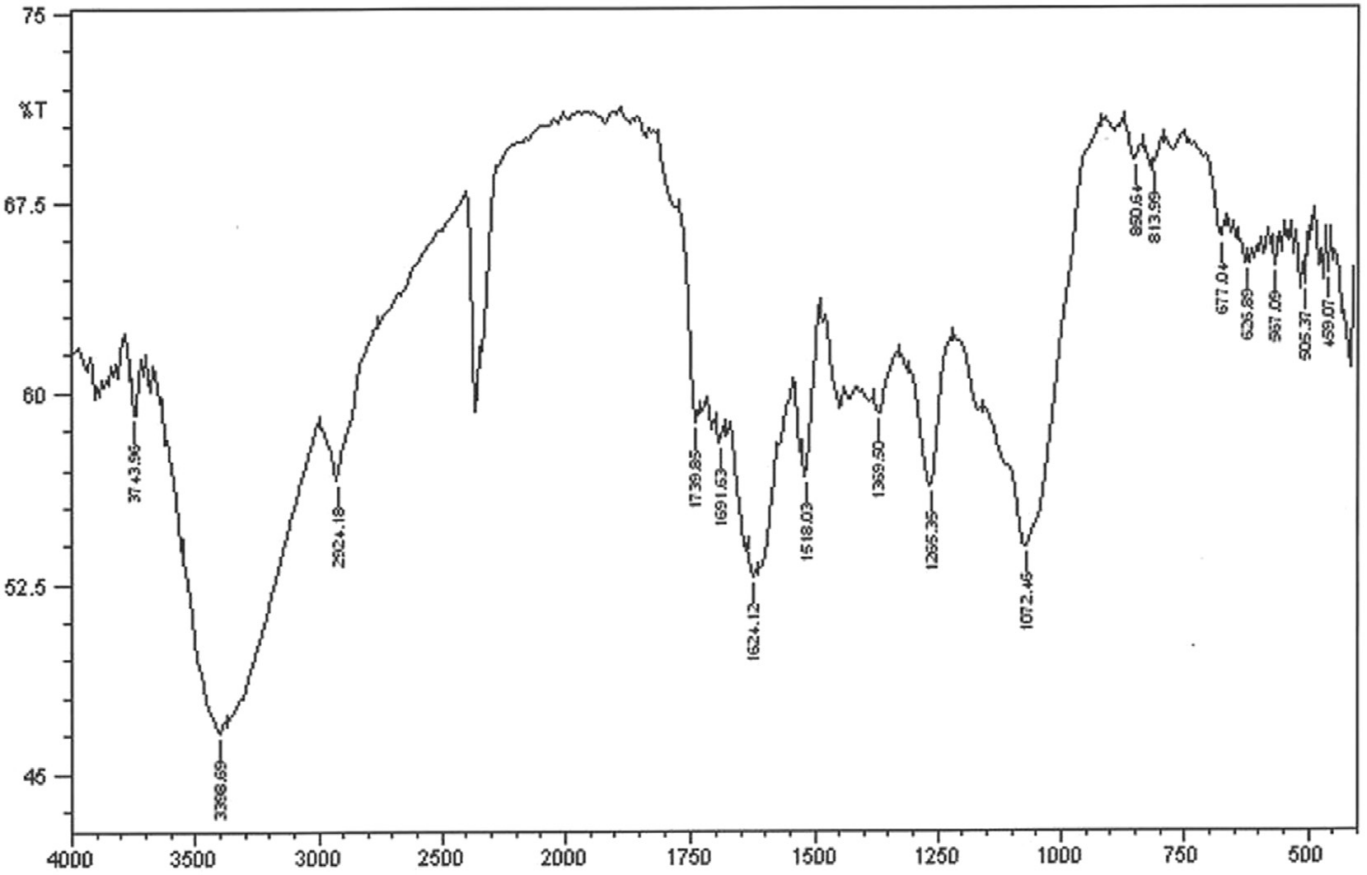
The FTIR spectrum of synthesized copper nanoparticles.

Results of FTIR analysis showed that the biomolecules in the extract reduced the copper sulfate solution; further, they would be used as coatings for nanoparticles. The bands at 3380, 2928, 1741, 1604, 1400, 1050, and 1271 cm^−1^ are attributed to the O–H stretching of alcohol and phenol, C–H stretching of the aliphatic group, C=O stretching of ester carbonyl, C=C stretching of the aromatic ring, and C–O stretching of ester, respectively (Fig. 3).

#### 3.1.3 SEM analysis

**Fig. 4:**
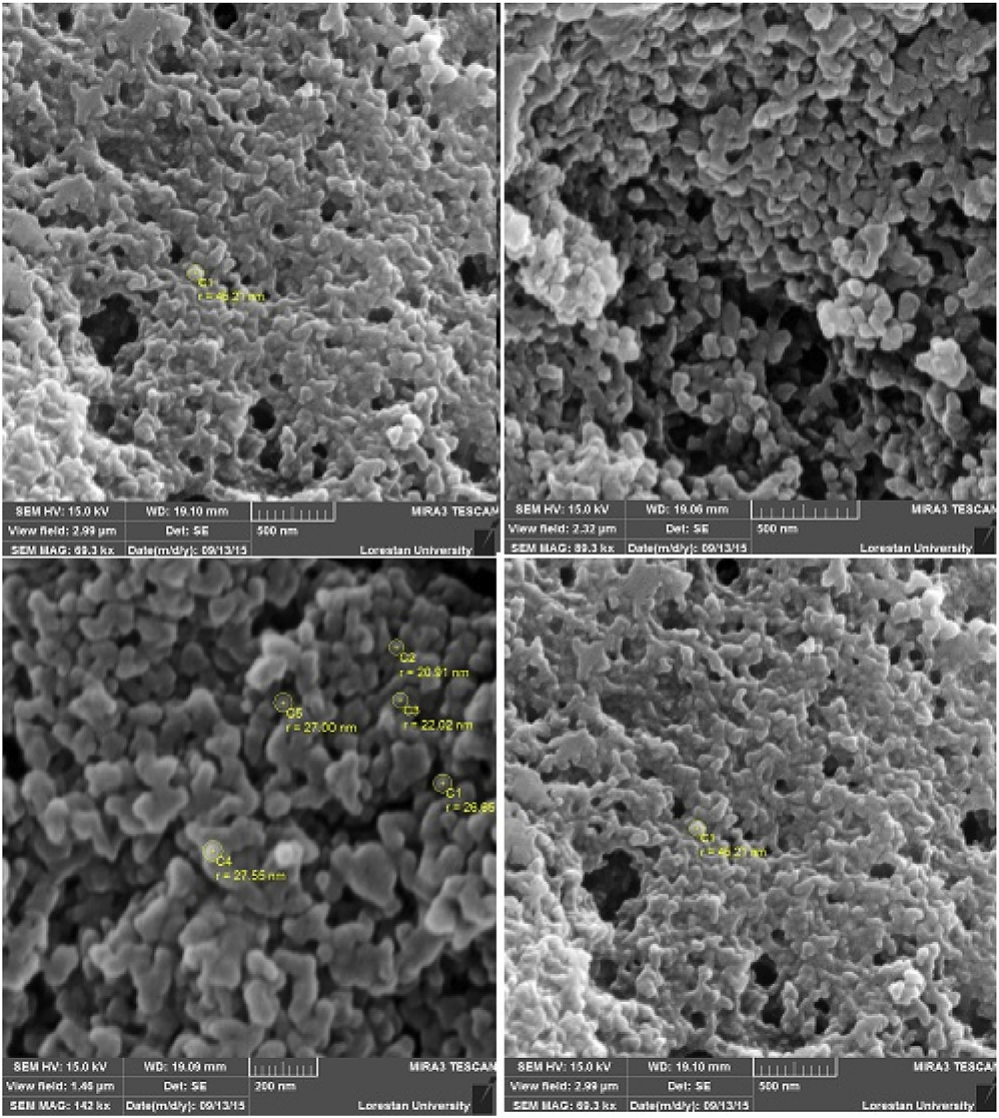
Scanning electron microscope of copper nanoparticles synthesized using aqueous extract of *Capparis spinosa* fruit.

Based on the obtained SEM results, the green synthesized CuNPs had a spherical morphology and the size of the particles was measured between 17 and 41 nm (Fig. 4).

### 3.2 The analgesic effect of CuNPs extract on tail flick test

**Fig. 5:**
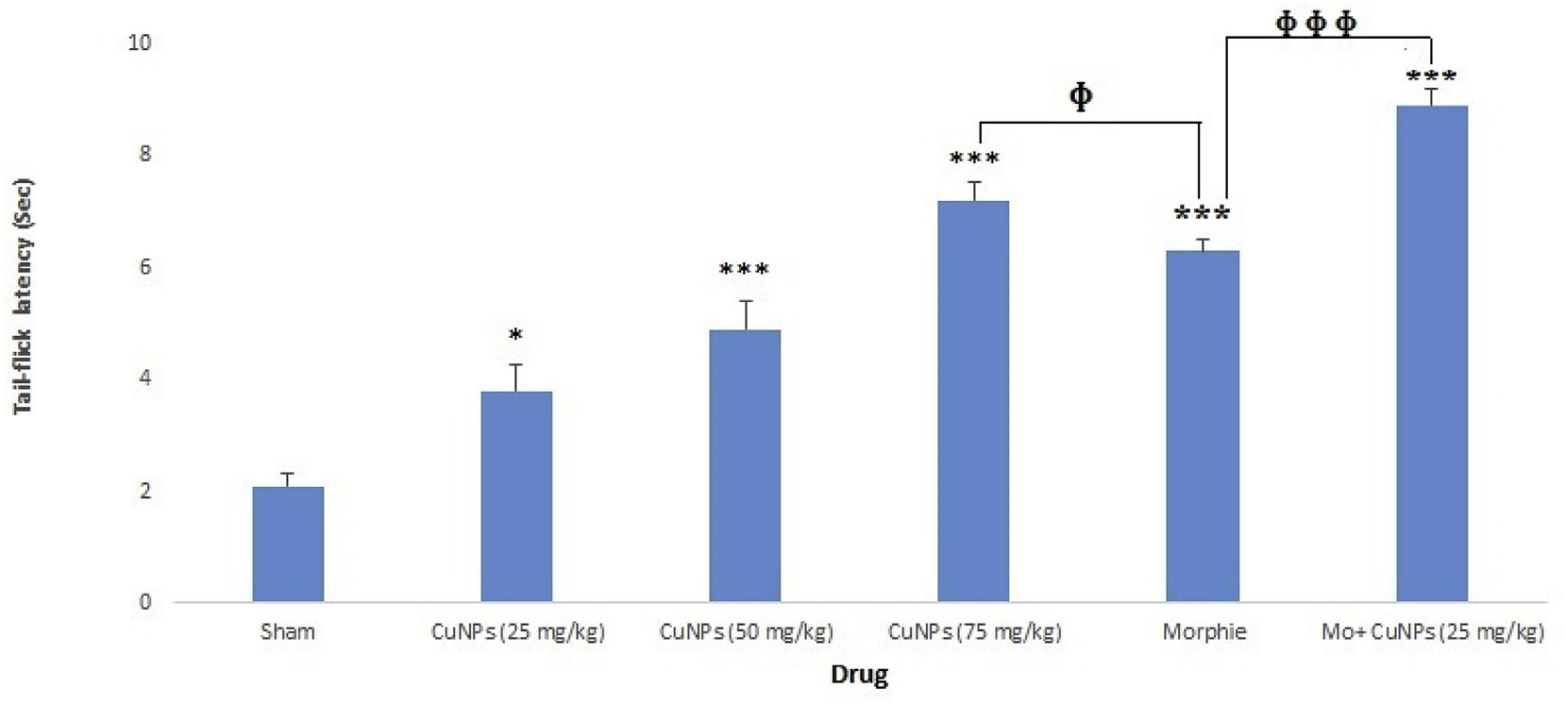
Effect of the CuNPs in various concentrations (25, 50, 75 mg/kg), morphine, and CuNPs (25 mg/kg) + morphine on the pain threshold of the mice in the tail-flick test. *p < 0.05, ***p < 0.001; ɸ p < 0.05; ɸɸɸ p < 0.001.

Fig. 5 shows the analgesic effects of CuNPs as the dose-dependent response in the tail-flick examination. CuNPs at the concentrations of 25, 50, and 75 mg/kg showed a mean latency time of 3.8, 4.9, and 7.2 s, respectively; demonstrating a significant (p < 0.05) antinociceptive effect in comparison with the control group. The results also showed that CuNPs at the dose 75 mg/kg and CuNPs (25 mg/kg) + morphine significantly (p < 0.05) increased the latency time compared with morphine alone.

### 3.3 The analgesic effect of CuNPs on the hot-plate test

**Fig. 6:**
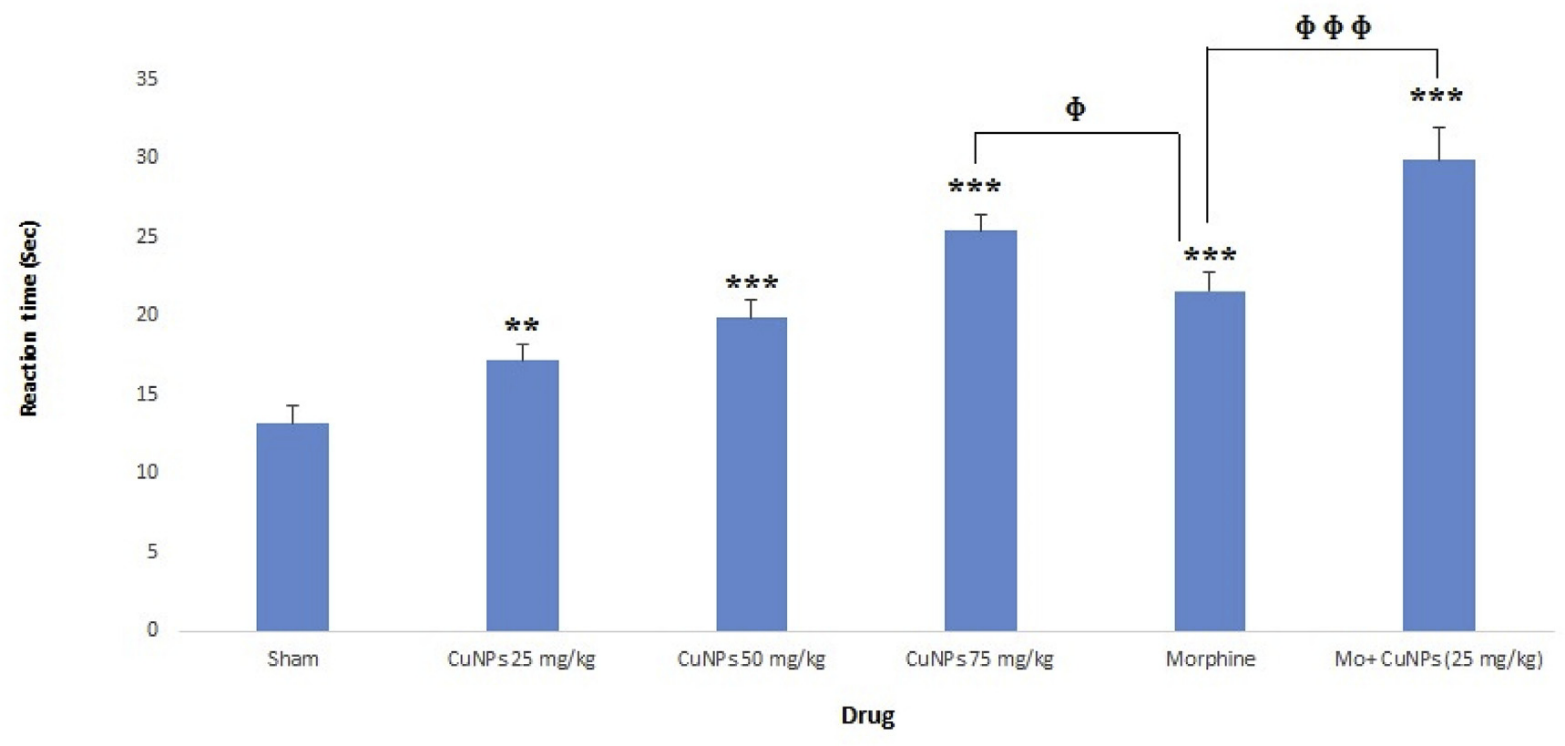
Effect of the CuNPs in various concentrations (25, 50, 75 mg/kg), morphine, and CuNPs (25 mg/kg) + morphine on the reaction time of the mice in the hot-plate test. *p < 0.05, ***p < 0.001; ɸ p < 0.05; ɸɸɸ p < 0.001.

As shown in Fig. 6, *CuNPs* at the concentrations of 25, 50, and 75 mg/kg had potent antinociceptive activity as a dose-dependent response. Based on the obtained results CuNPs at the dose 75 mg/kg and CuNPs (25 mg/kg) + morphine showed a longer reaction time compared with morphine alone.

### 3.4 Rotarod test

Based on the obtained results by motor coordination test, no significant (p > 0.05) difference was observed after the administration of CuNPs at the doses of 25, 50, and 75 mg/kg in the sensory-motor test.

## 4 Discussion

Pain is a sensory and protective mechanism that makes the living organism duly aware of the harmful factors of the periphery and respond accordingly [21]. Since all people with any level of life have the earliest age experience of pain perception, achieving ways to reduce pain has been constantly one of the most important requirements of human life [4]. Although humans with discovering opioid drugs have achieved this demand to a great extent, the side effects such as tolerance to analgesia, addiction, respiratory depression, sleep apnea, fatigue, and vomiting have raised a serious problem with taking these medications [4].

Attaining to new agents that despite being low-risk have the desired properties caused special attention of researchers to “nanotechnology”. According to scientific literature, nanotechnology is the ability to produce new materials, tools, and systems with control over molecular and atomic levels. This science, which many researchers believe will have a huge impact on the future of humankind, is applicable in many fields such as pharmacology, medicine, the chemical industry, mechanics, cosmetics, resource sustainability, and transportation [5].

Although physical and chemical methods may have good biological activity, due to cost-intensiveness, difficulty in supplying, and, above all, high toxicity, today they have been replaced by more favorable methods such as Green Synthesis. Green Synthesis is the design of chemical products and processes that reduce or eliminate the use of the generation of hazardous substances [22].

Instinctively, from the beginning of history, humans have been searching for herbal resources to find drugs to treat their diseases. *C. spinosa*, which is known among the Persians with the local name “Kabar”, is an acanaceous and full of foliage shrubs with numerous medicinal properties, including diuretics, diaphoretic, laxatives, and anti-worms [23]. In this study, we used *C. spinosa* fruits as a biological agent to facilitate the synthesis of CuNPs and, subsequently, examined its acute and chronic analgesic effects on laboratory animals. In this investigation, using this plant and in the form of green synthesis methods, we tried to obtain CuNPs; the synthesized CuNPs were spherical and their approximate size was between 17 and 41 nm. Until now, plants such as *Euphorbia nivulia* [24], *Magnolia kobus* [25], *Nerium oleander* [26], and *Eclipta prostrata* have been used in the biosynthesis of CuNPs. Nevertheless, recognizing the plants’ capacity as biological material for the synthesis of nanoparticles in detail requires more studies [[27], [28], [29]].

To evaluate the analgesic effects of the intended experimental factors, the hot plate is one of the most reliable tests. Based on the obtained data, it can be stated that the synthesized CuNPs may act as a dose-dependent response and reduce the pain caused by thermal stimulation. In this test, there was a significant difference between the groups receiving CuNPs and the control group in terms of being affected by the pain (p < 0.05). Another test that was used to study spinal responses and decipher the central analgesic route was the tail-flick test; the possible conjecture is that the analgesic effects of CuNPs are applied via the central nervous system.

So far, several studies have been conducted on the antinociceptive effects of biosynthetic nanoparticles. For example, Islam et al. (2015) in a study in Pakistan concluded that gold nanoparticles improve the therapeutic ability of *Euphorbia milii* extract and have considerable antinociceptive effects, muscle relaxant, and sedative assets [30]. In Iran, Jahangiri et al. (2013) investigated the effects of magnesium oxide nanoparticles on the perception of the chemical pain resulting from the two tests of acetic acid and formalin in NMRI mice. The results of this study showed that Mgo-NPs affect the mechanisms involved in pain and possibly affect the analgesic properties through blockage of NMDA glutamate receptors [31].

In another similar study in Iran, Kesmaty et al. (2012) examined the antinociceptive effects of zinc oxide nanoparticles on rats. The results showed significant ZnO-NPs analgesic effects at low levels (0.5 and 1 mg/kg) compared to the control group in the Tail flick test. Also, the results of the hot plate test at low levels ZnO-NPs (0.5 mg/kg) indicated the antinociceptive effects of this nanoparticle [32]. de Araújo et al. (2017) also have demonstrated that gold nanoparticles showed peripheral analgesia in the hot plate test at the dose of 1500 μg/kg in the tested Swiss mice [33].

Chiguvare et al. (2016) showed that the Buchu plant extract along with silver nanoparticles showed potent analgesic effects in Swiss albino mice in formalin test compared with the aspirin drug and the extract alone [34]. These differences in efficacy and analgesic activity of nanoparticles can be related to the type of nanoparticles, method of preparation as well as type of studied analgesic examination.

To the best of our knowledge, there is no article about the analgesic effects of CuNPs synthesized by chemical and biological methods, however, there are some studies that prove their anti-microbial and antioxidant effects [23]. In this regard, in some scientific texts, the antinociceptive properties of the copper element and its role in the mechanism of pain control have been mentioned [6]. Although the exact knowledge of the mechanism of the antinociceptive effect of CuNPs requires further studies, we may relate this effect to the central mechanisms.

## 5 Conclusion

The results of present study confirm the antinociceptive effects of biosynthetic copper nanoparticles from *C. spinosa* fruits. Although it is clear that the use of it as a pain reliever requires additional researches in the field of pharmacology and toxicology. In addition, knowing the exact physiological mechanisms involved can also be a major step towards finding more effective drugs to relieve pain.

## Ethical approval

No require.

## Sources of funding

No funding.

## Author contribution

SN: Writing, Supervisor.

SJ: Experiment, data collection.

MN: Experiment, data collection.

KE: Experiment, data analysis.

HM: Experiment, study design.

SS: Experiment, data analysis.

MK: study design.

## Consent

No need.

## Registration of research studies

Name of the registry:

Unique Identifying number or registration ID:

Hyperlink to the registration (must be publicly accessible):

## Guarantor

Sedigheh Nadri.

## Provenance and peer review

Not commissioned externally peer reviewed.

## Declaration of competing interest

The authors declare no conflict of interest in this work.
